# Postnatal outcome of fetal hydronephrosis: Implications for prenatal counselling

**DOI:** 10.4103/0970-1591.60446

**Published:** 2010

**Authors:** Ramesh Babu, Venkata Sai

**Affiliations:** Department of Pediatric Urology, Sri Ramachandra Medical College and Research Institute, Chennai, India; 1 Radiology, Sri Ramachandra Medical College and Research Institute, Chennai, India

**Keywords:** Antenatal counselling, fetal ultrasound, hydronephrosis

## Abstract

**Objectives:**

Hydronephrosis is commonly detected during antenatal scans. There are multiple conflicting prognostic factors in the literature with no clear focus on the postnatal outcome. The aim of the study is to assess the outcome of fetal hydronephrosis, based on antenatal sonography.

**Materials and Methods:**

Based on the third trimester fetal ultrasound findings, patients were divided into group I (unilateral hydronephrosis) and group II (bilateral hydronephrosis, ureteric dilatation, bladder thickening, etc). Postnatal evaluation and follow-up was performed by a single physician with uniform protocol. The outcomes, spontaneous resolution vs. surgical intervention, were compared between groups. Among group I, further analysis of outcome was done based on 32-week fetal pelvic antero posterior diameter (APD).

**Results:**

Among a total of 116 patients in the study group; group I had 78 patients, 7 (9%) required surgery; group II had 38 patients, 21(55%) required surgery. The difference in outcome between the groups was statistically significant (*P* = 0.002). Among those with unilateral hydronephrosis, none (0/55) with APD <15 mm required surgery, while all patients (4/4) with fetal APD> 30 mm required surgery. In those with APD between 15-30 mm, 3/19 required surgery and prolonged follow-up was required to arrive at the decision. The difference in outcome between the subgroups was statistically significant (P< 0.001, Chi-square test).

**Conclusions:**

The results of our study show that simple unilateral fetal hydronephrosis runs a benign course. In the presence hydronephrosis larger than 15 mm, bilateral disease, or bladder distension, detailed postnatal evaluation and regular follow-up is warranted to plan a timely intervention. The above data could be used in prenatal counselling of these parents. Further larger studies are warranted to through more evidence.

## INTRODUCTION

Hydronephrosis is one of the common anomalies detected during a fetal ultrasound.[1] Of late counselling for antenatal hydronephrosis has gained popularity and more and more parents attend these clinics to find out the outcome.[2] Although antenatal ultrasound has revolutionized the management of renal anomalies, especially in identifying the ones which require early intervention; it has also unmasked a vast majority of neonates who otherwise are ‘not patients’ in the conventional sense with clinical symptoms and signs.[3] Prenatal ultrasound identifies the whole spectrum including many children from the milder end, thereby exposing them to excessive imaging both pre and postnatally.[4] In addition, the parents of these ‘mild spectrum’ fetuses are put through undue agony during pregnancy and later.

To differentiate between an abnormality of the urinary tract that is clinically important from the less significant one is of utmost important for the physician involved in counselling of these parents.[5] This can only be achieved by assessing the postnatal outcome of the renal anomalies picked up on maternal ultrasound. The existing literature contains multiple conflicting parameters to predict the outcome of antenatally detected hydronephrosis. [6–10] The aim of the study is to assess the outcome of fetal hydronephrosis, segregate them into simple groups so that the milder ones could be favourably counselled. At the same time, patients with more serious type could be cautioned regarding regular follow-up, prompt evaluation and timely intervention whenever necessary. The data from this study should be useful for the physicians involved in the counselling of these parents pre and postnatally.

## MATERIALS AND METHODS

All the patients who were diagnosed with antenatal hydronephrosis in our hospital during January 2003 December 2006 were included. Patients were followed up from third trimester of pregnancy to a variable period (1-4 yrs) after birth. Patients who had ultrasound at multiple centres or those defaulted for follow-up were excluded. All ultrasonograms were done by a single sonologist. Postnatal evaluation was uniformly done and the decision for intervention was made by a single pediatric urologist.

Postnatal evaluation included ultrasonogram, voiding cysto urethrogram, and nuclear renogram. A diagnosis of transient hydronephrosis was made when the hydronephrosis resolved on the first or subsequent postnatal evaluations: Ultrasonogram or nuclear renograms. Table 1 summarizes the diagnoses and outcomes.

**Table 1:** Etiology of fetal hydronephrosis and outcome after postnatal evaluation. In group I (isolated unilateral fetal hydronephrosis) and group II (bilateral fetal hydronephrosis, ureteric dilatation or bladder wall thickening)

Groups	Diagnosis	Total number	Surgical intervention
Group I	Transient hydronephrosis	71	Nil
	Uretero pelvic junction obstruction	7	7
Group II	Vesico ureteric reflux	22	5
	Posterior urethral valves	12	12
	Lower ureteric obstruction	4	4
Total	All diagnoses	116	28

Based on the 32-week fetal ultrasound findings, patients were divided into two groups: Group I (unilateral hydronephrosis) and group II (bilateral hydronephrosis, ureteric dilatation, bladder thickening, etc). Based on the existing literature, [6–10] group I was further divided into three subgroups depending on the 32-week fetal pelvic antero posterior diameter (APD): <15, 15-30, and > 30 mm. The outcomes, spontaneous resolution vs. surgical intervention, were compared between groups. Statistical analysis was done using Fisher's exact test and Chi-square test and the difference in outcome was considered significant when the *P* value was less than 0.05.

## RESULTS

**Figure 1:**
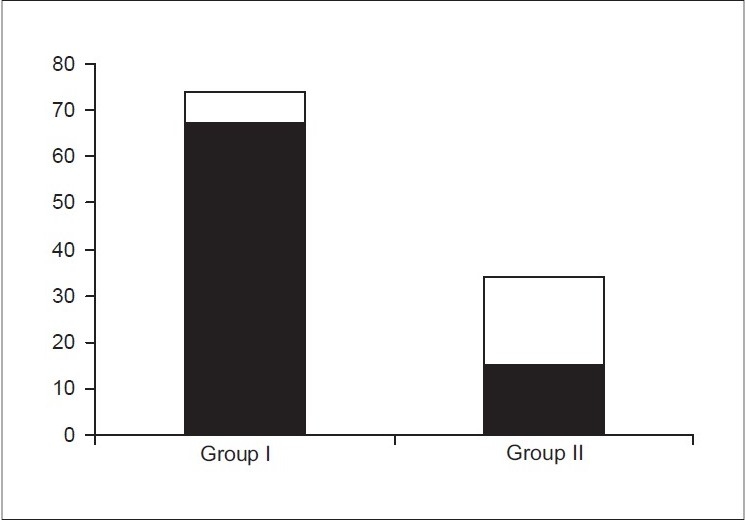
Postnatal outcome of fetal hydronephrosis. Shaded part represents spontaneous resolution and white part represents surgical intervention. In group I (isolated unilateral fetal hydronephrosis) 7/78 (9%) only required surgery, while in group II (bilateral fetal hydronephrosis, ureteric dilatation or bladder wall thickening) 21/38 (55%) required surgery

Among a total of 140 patients registered, 24 were excluded (ultrasound at different centres and lack of follow-up), leaving 116 in the study group. Group I had 78 patients and only 7 (9%) required surgery. Group II had 38 patients and 21 (55%) required surgery. The difference in outcome between the groups [Figure 1] was statistically significant (*P* = 0.002 Fisher's exact test).

Table 2 summarizes the subgroups in group I. All patients (4/4) with fetal APD > 30 mm required surgery, while none (0/55) with APD < 15 mm required surgery. In those with APD between 15-30 mm, 3/19 required surgery and prolonged follow-up was required to arrive at the decision. The difference in outcome between the subgroups was statistically significant (P< 0.001, Chi-square test).

**Table 2:** Outcome in group I (isolated unilateral fetal hydronephrosis) based on pelvic antero posterior diameter determined at 32-week fetal ultrasound

Fetal pelvic AP diameter	Total number	Surgery required
<15 mm	55	Nil
15-30 mm	19	3
>30 mm	4	4
Total	78	7

## DISCUSSION

With the advent of routine obstetric ultrasound, a vast majority of renal anomalies are picked up on antenatal ultrasound. The incidence of antenatally diagnosed hydronephrosis is as high as 1-5%.[1–5] Antenatal diagnosis of hydronephrosis causes significant distress to the parents during pregnancy. The term hydronephrosis although is clearly descriptive, it does not by itself mention the underlying etiology such as uretero pelvic junction obstruction, vesico ureteric reflux, posterior urethral valve, etc.

For a treating physician, in addition to the etiology, it is also essential to know the natural history of the disease. On the other hand, for the parents, obstetricians, pediatricians, and urologists who are involved in the antenatal counselling, it would be easy if the fetal hydronephrosis outcomes are explained in a clear and simple way based on the sonographic findings rather than the potential differential diagnoses.

Lee *et al*.[5] in their meta analysis on the outcome of antenatal hydronephrosis, have concluded that children with any degree of antenatal hydronephrosis are at a greater risk of postnatal pathology compared to normal population. However, they could not use a single fetal ultrasound criterion as a predicting factor due to lack of conformity in reporting. Onen[6] questioned the usefulness of APD and Society for Fetal Urology grading of hydronephrosis and proposed a new grading system. Mallik and Watson[7] reported a trend towards larger renal pelvic APD on third trimester scans being associated with more significant pathology, but cautioned a lot of clinical overlap and highlighted the need for a cautions antenatal counselling. The existing literature [8–15] does not provide a clear picture on clinical relevance of detecting fetal hydronephrosis and its value in predicting postnatal outcome.

Studies on outcome of fetal hydronephrosis could be complex with overlapping of multiple diagnoses. Although we did not encounter patients with bilateral pelviureteric junction (PUJ) or multiple pathologies such as vesicoureteral reflux (VUR) and PUJ in the same patient, these are likely to complicate outcome assessment. In addition, observer variation between sonologists and lack of uniform protocol or multiple surgeons deciding for surgical intervention could bias the results. We acknowledge that our numbers are small, however, they could act as a pilot study for a larger well-planned study.

The results of our study show that simple unilateral hydronephrosis, detected prenatally, runs a benign course. The chances of spontaneous resolution are high, especially when the 32-week fetal pelvic APD is less than 15 mm. These data could be used in counselling parents of such foetuses favorably. On the other hand, in the presence of hydronephrosis >15 mm, bilateral disease, ureteric dilatation or bladder distension on prenatal ultrasound, detailed evaluation and prolonged follow- up is warranted to decide on a timely intervention. Larger prospective studies with well-defined prenatal screening protocols and uniform postnatal follow-up are warranted to address the outcome of fetal hydronephrosis.
